# Multi-Objective Task Scheduling for Vehicle–UAV Synchronous Cooperative Distribution Network Inspection

**DOI:** 10.3390/s26103122

**Published:** 2026-05-15

**Authors:** Xiaoyi Liu, Yuhan Yin, Kunxiao Wu, Yetong Zhang, Jianyong Zheng, Fei Mei

**Affiliations:** 1 School of Electrical Engineering, Southeast University, Nanjing 210096, China; 220255278@seu.edu.cn (X.L.); yinyuhan@seu.edu.cn (Y.Y.); 220243168@seu.edu.cn (K.W.); 220255288@seu.edu.cn (Y.Z.); 2 College of Energy and Electrical Engineering, Hohai University, Nanjing 211100, China; meifei@hhu.edu.cn

**Keywords:** unmanned aerial vehicle, vehicle–UAV cooperative inspection, distribution line, multi-objective optimization, deep reinforcement learning

## Abstract

To address the challenges of significant vehicle parking constraints, limited UAV endurance, and insufficient multi-task coordination efficiency in distribution network inspection, this paper proposes a vehicle–UAV synchronous cooperative inspection task scheduling method based on multi-objective twin delayed deep deterministic policy gradient and nondominated sorting genetic algorithm II (MOTD3-NSGA-II). First, a vehicle–UAV synchronous cooperative inspection model is established by considering staged vehicle repositioning, same-site UAV launch, landing, and retrieval, as well as state-of-charge constraints. On this basis, a multi-objective optimization model is formulated with task coverage, mission completion time, minimum residual state of charge, and load balance as objectives. Then, a bi-level closed-loop solution framework is developed, in which NSGA-II is employed to optimize cooperative parameters and objective preference weights, while the inner-layer MOTD3 learns UAV scheduling policies in a continuous action space. Finally, the proposed method is validated in four simulation scenarios with different task scales and spatial distribution characteristics. The results show that 100% task coverage is achieved in all four scenarios, with mission completion times of 11,109 s, 9693 s, 10,538 s, and 10,721 s, respectively, while the minimum residual state of charge is maintained within 0.28–0.36. The results demonstrate that the proposed method can balance inspection completeness, execution efficiency, energy safety, and cooperative stability, providing a useful reference for intelligent task scheduling in vehicle–UAV cooperative distribution network inspection.

## 1. Introduction

Distribution network inspection is a critical component for ensuring the safe operation of line equipment, enhancing condition awareness, and supporting refined operation and maintenance. With the continuous expansion of distribution networks, the increasing diversity of equipment types, and the growing spatial span of inspection tasks, traditional inspection approaches relying on manual pole climbing, visual inspection, or single ground-based equipment can no longer simultaneously meet the requirements of efficiency, safety, and refined operation and maintenance. Owing to their strong maneuverability, flexible deployment, low operational risk, and rich inspection perspectives, unmanned aerial vehicles (UAVs) have been widely applied in recent years to the inspection of transmission lines, distribution lines, and high-voltage equipment. Existing studies have explored UAV-based power inspection from the perspectives of review studies, key technical systems, UAV-LiDAR-assisted inspection, autonomous inspection systems for high-voltage transmission lines, and intelligent monitoring of power lines [1,2,3,4,5]. Among them, Ref. [3] applied UAV-LiDAR to intelligent inspection of power facilities, improving the acquisition capability of spatial information for lines and equipment, while Ref. [4] designed an autonomous UAV inspection system for high-voltage transmission lines, promoting the development of UAV inspection from assisted imaging toward autonomous operation. However, the above studies mainly focus on single-UAV inspection, object detection, scene perception, or system implementation, and pay insufficient attention to the multi-stage cooperative task scheduling problem when vehicles are introduced as mobile support platforms.

Considerable effort has also been devoted to autonomous flight and path planning in UAV-based power inspection, including power line detection and tracking, vision-assisted landing, safety-distance-constrained path planning, reinforcement-learning-based inspection decision-making, and general path planning and obstacle avoidance [6,7,8,9,10]. Ref. [6] proposed an autonomous power line detection and tracking system for UAVs, enhancing their ability to identify and follow line targets, while Ref. [8] developed a UAV inspection path-planning method with safety-distance constraints for substation inspection scenarios, which helps improve path safety. Overall, such methods can improve UAV autonomous perception, path generation, and obstacle avoidance in local inspection scenarios. Nevertheless, most of them still focus on the flight path or local decision-making process of the UAV itself. For large-scale distribution network inspection tasks, inspection points are usually discretely distributed along lines, roads, and regional boundaries. A single UAV is often unable to accomplish the global task independently due to endurance limitations, and system efficiency is jointly affected by vehicle parking locations, UAV return-and-recharge operations, and task-stage transitions. Therefore, addressing inspection tasks solely from the perspective of single-UAV path planning is insufficient to fully characterize the coupling relationships among vehicle repositioning, UAV launch and retrieval, task allocation, and energy constraints.

To address UAV path planning and dynamic obstacle avoidance in complex environments, existing studies have further considered methods such as multi-objective inspection, the integration of sampling-based planning and local obstacle avoidance, and improved dynamic window approaches [11,12,13]. Among them, Ref. [12] proposed a UAV dynamic path-planning method integrating improved Informed-RRT* with the dynamic window approach, thereby improving path search and obstacle avoidance in complex environments, while Ref. [13] proposed a dynamic adaptive dynamic window approach, enhancing local obstacle avoidance performance in dynamic environments. Although these studies offer advantages in improving path search efficiency, dynamic obstacle avoidance capability, and flight trajectory feasibility, they still mainly belong to the category of flight-path-level planning. In contrast, vehicle–UAV cooperative power grid inspection must address not only how the UAV should fly, but also when the vehicle should arrive at a parking point, from which parking point the UAV should take off and be retrieved, how tasks should be allocated across different stages and UAVs, and how the system should balance efficiency and energy safety. Therefore, such problems are essentially multi-objective optimization problems involving the coupled interactions of path planning, task allocation, energy management, and cooperative scheduling.

Vehicle–UAV cooperative routing provides a new technical paradigm for large-scale task execution. In recent years, problems such as truck–drone routing, cooperated trucks and drones routing, truck–drone team logistics, and dynamic synchronization routing have attracted extensive attention [14,15,16,17,18]. Related studies have shown that vehicle routes, UAV routes, and the spatiotemporal synchronization relationship between them can significantly affect overall system efficiency. For example, Ref. [15] proposed a heuristic multi-drop route-planning method for truck–drone team logistics, which can coordinate the service process of vehicles and UAVs in multi-delivery-point tasks, while Ref. [17] further proposed a reliable truck–drone routing model with dynamic synchronization, improving the robustness of vehicle–UAV cooperative routing under uncertainty. However, most existing vehicle–UAV cooperative studies are oriented toward logistics delivery or general service-routing scenarios, with optimization objectives typically emphasizing delivery time, transportation cost, or customer service efficiency. In contrast, distribution network inspection tasks involve stronger safety constraints and more distinctive engineering–operational characteristics, such as inspection points discretely distributed along lines, vehicles being allowed to stop only at limited parking points, UAVs needing to maintain sufficient return-flight energy, and the requirement for task completeness and equipment recoverability during inspection. Therefore, existing vehicle–UAV cooperative routing models are difficult to directly apply to distribution network inspection scenarios involving fixed parking-point support, same-site UAV launch and retrieval, stage-wise vehicle waiting, and SOC safety constraints.

From the perspective of cooperative mechanisms, vehicle–UAV cooperation can generally be divided into asynchronous cooperation and synchronous cooperation. Asynchronous cooperation allows vehicles and UAVs to advance tasks in parallel and can theoretically help reduce the total execution time, but it also significantly increases the difficulty of communication maintenance, cross-point retrieval, real-time rescheduling, abnormal-event handling, and safety supervision. Especially in distribution network inspection scenarios, where task points are often distributed along line corridors, road boundaries, or complex outdoor environments, if UAVs perform cross-point retrieval or dynamic rendezvous while the vehicle is repositioning, communication delays, localization errors, wind disturbances, or estimation errors in remaining battery energy may lead to return failure, retrieval difficulty, or task interruption. Existing studies on multi-agent cooperative control have shown that communication delays, asynchronous interactions, and cooperation–competition network relationships can significantly affect system consistency and cooperative stability [19,20]. In contrast, synchronous cooperation requires UAVs to take off from the current parking point and return to the same parking point, while the vehicle moves to the next stage only after all relevant UAVs in the current stage have been retrieved. Although this mode may sacrifice part of the time efficiency, it can improve UAV retrieval reliability, controllability of battery management, supervisability of task stages, and engineering safety, making it more suitable for safety-priority distribution network inspection tasks. Therefore, this paper focuses on the vehicle–UAV synchronous cooperative inspection mode.

In terms of solution methods, deep reinforcement learning and multi-objective evolutionary optimization provide effective tools for complex cooperative scheduling problems. TD3 alleviates value overestimation and training oscillation in continuous control through the twin-Critic structure, target policy smoothing, and delayed policy update mechanism [21]. Related studies based on TD3 and its multi-agent extensions have been applied to multi-UAV autonomous path planning and local path decision-making scenarios [22,23]. On the other hand, NSGA-II can obtain a diverse Pareto solution set among multiple conflicting objectives through fast nondominated sorting and the crowding-distance mechanism [24]. However, a single reinforcement learning method usually relies on fixed reward weights and cannot directly generate diversified scheduling schemes under different objective preferences, whereas a single multi-objective evolutionary algorithm has difficulty effectively handling dynamic state feedback and policy learning problems in continuous action spaces. For vehicle–UAV synchronous cooperative inspection tasks, the system needs not only to achieve global trade-offs among task coverage, mission completion time, minimum residual SOC, and load balance, but also to learn continuous policies such as UAV target selection, speed adjustment, and return decision-making during task execution. Therefore, it is necessary to develop a bi-level optimization framework that simultaneously accounts for multi-objective global search and continuous policy learning.

In summary, important progress has been made in UAV-based power inspection, UAV path planning, vehicle–UAV cooperative routing, multi-agent cooperative control, and intelligent optimization algorithms. However, the safety-priority synchronous inspection scenario in distribution networks still faces the following limitations: first, insufficient modeling of cooperative scheduling under fixed vehicle parking, same-site UAV launch and retrieval, and stage-wise vehicle waiting; second, insufficient consideration of the coordination among multiple objectives, including task coverage, mission completion time, minimum residual SOC, and load balance; and third, the difficulty of existing single-layer reinforcement learning or single-layer multi-objective optimization methods in simultaneously addressing continuous-action decision-making and Pareto multi-objective trade-offs. To address these issues, this paper proposes a vehicle–UAV synchronous cooperative power grid inspection task scheduling method based on Multi-Objective Twin Delayed Deep Deterministic Policy Gradient and Nondominated Sorting Genetic Algorithm II (MOTD3–NSGA-II).

The main contributions of this paper are summarized as follows:

1. A vehicle–UAV synchronous cooperative task scheduling model for distribution network inspection is proposed. This model incorporates stage-wise vehicle repositioning, fixed parking-point support, same-site UAV launch and retrieval, task completion criteria, and SOC constraints into a unified framework, and characterizes the coupling relationships among vehicle waiting, UAV stage-wise execution, and energy safety under the synchronous cooperative mode.
2. A bi-level multi-objective optimization method based on MOTD3–NSGA-II is proposed. The outer-layer NSGA-II is used to search cooperative parameters and multi-objective preference weights, while the inner-layer MOTD3 is used to learn UAV scheduling policies in a continuous action space, thereby achieving joint optimization of task coverage, mission completion time, minimum residual SOC, and load balance.
3. A multi-scenario simulation-based validation framework is constructed to analyze the vehicle–UAV synchronous cooperative inspection process under different task scales and spatial distribution conditions. Through results including vehicle–UAV cooperative trajectories, task completion progress, SOC evolution, and overall performance indicators, the proposed method is validated in terms of inspection completeness, execution efficiency, and energy safety under the prescribed synchronous cooperative scenarios.

## 2. Problem Description and Model Formulation

### 2.1. Problem Description

Under typical operating conditions, distribution network inspection tasks are generally characterized by discrete task-point distribution, large spatial span, restricted road accessibility, and limited endurance of a single UAV. When a ground vehicle is introduced as a platform for UAV transport, deployment, retrieval, and recharging, the problem is no longer a single-UAV path-planning problem, but instead evolves into a multivariable cooperative scheduling problem involving vehicle parking, UAV launch and retrieval, task allocation, energy management, and stage transition.

This paper investigates a vehicle–UAV synchronous cooperative power grid inspection scenario. In this scenario, the vehicle sequentially travels to each parking point along a predetermined road network and completes the unified deployment and retrieval of UAVs at each parking point. After taking off from the current parking point, multiple UAVs inspect the task points assigned to the current stage. The vehicle does not depart for the next parking point until all tasks in the current stage have been completed and all relevant UAVs have returned. Therefore, the vehicle movement process and the UAV operation process exhibit significant stage-wise coupling in the temporal dimension. The vehicle waiting time, the task partitioning strategy among UAVs, and the battery consumption process jointly affect the overall inspection efficiency and operational safety of the system.

Based on the above characteristics, this paper establishes a unified model of the vehicle–UAV synchronous cooperative inspection process from the perspectives of the task-point set, parking-point set, onboard UAV states, vehicle parking process, task completion criteria, and energy constraints. On this basis, a multi-objective optimization model is formulated with task coverage, mission completion time, minimum residual state of charge, and load balance as the core performance indicators, thereby providing a theoretical foundation for the subsequent design of the bi-level MOTD3–NSGA-II optimization method.

### 2.2. Basic Definitions

The set of inspection task points is defined as:

(1) $$T=\left\{\tau_{1},\tau_{2},\cdots ,\tau_{N}\right\}$$

The spatial coordinates of the *n*-th task point are denoted by:

(2) $$q_{n}={\left[x_{n},y_{n},z_{n}\right]}^{T},\;n=1,2,\cdots ,N$$

The set of parking points available to vehicle *c* is defined as:

(3) $$P=\left\{p_{1},p_{2},\cdots ,p_{M}\right\}$$

The position of the *k*-th parking point is denoted by:

(4) $$p_{k}={\left[x_{k}^{c},y_{k}^{c},z_{k}^{c}\right]}^{T},\;k=1,2,\cdots ,M$$

The set of onboard UAVs is defined as:

(5) $$U=\left\{u_{1},u_{2},\cdots ,u_{K}\right\}$$

At discrete time t=0,1,…,T, the position of the vehicle is denoted by:

(6) $$r_{c}(t)={\left[x_{c}(t),y_{c}(t),z_{c}(t)\right]}^{T}$$

The position of UAV *i* is denoted by:

(7) $$r_{i}(t)={\left[x_{i}(t),y_{i}(t),z_{i}(t)\right]}^{T},\;i=1,2,\cdots ,K$$

The task completion state variable is defined as:

(8) $$y_{n}(t)\in \{0,1\}$$

In the equation: if task point $\tau_{n}$ has been successfully inspected before time *t*, then $y_{n}(t)=1$; otherwise, $y_{n}(t)=0$. Therefore, the number of completed tasks at time *t* is:

(9) $$N_{\mathrm{fin}}(t)=\sum_{n=1}^{N}y_{n}(t)$$

Accordingly, the task coverage rate is

(10) $$\rho (t)=\frac{1}{N}\sum_{n=1}^{N}y_{n}(t)$$

To characterize the correspondence between task points and parking stages, a binary variable is introduced as:

(11) $$x_{nk}\in \{0,1\}$$

In the equations: $x_{nk}=1$ denotes that task point $\tau_{n}$ is assigned to parking stage *k*. To ensure that each task point is processed in only one stage, the following constraint should be satisfied:

(12) $$\sum_{k=1}^{M}x_{nk}=1,\;\forall n=1,2,\cdots ,N$$

### 2.3. Vehicle Parking and UAV Operation Model

The same-site launch and retrieval constraint is used to characterize a safety-priority synchronous cooperative inspection mechanism; UAVs take off from the current parking point of the vehicle and return to the same parking point after completing the inspection tasks of the current stage, and the vehicle proceeds to the next parking stage only after all relevant UAVs have been successfully retrieved. Compared with asynchronous cooperation, cross-site retrieval, or dynamic rendezvous modes, this setting is more conservative, but it can improve UAV retrieval reliability, controllability of SOC management, and supervisability of stage-wise operations. Therefore, the proposed model is mainly applicable to distribution network inspection scenarios in which the task points and candidate parking points are relatively well defined, the vehicle is required to provide stage-wise support for UAV operations, and high requirements are imposed on safety and recoverability.

Furthermore, in this study, the vehicle transfer time is estimated based on the shortest-path distance in the road network and the average vehicle speed. This formulation is mainly used to establish the temporal coupling relationship between staged vehicle relocation and UAV operation waiting time. It does not further consider practical factors such as traffic congestion, temporary road closures, changes in road conditions, vehicle acceleration and deceleration processes, or on-site traffic control. Therefore, the vehicle transfer results mainly reflect the scheduling time cost under the predefined road network and average-speed assumptions, rather than an accurate prediction of travel time in complex real-world road environments.

In vehicle–UAV synchronous cooperative inspection, the vehicle sequentially visits each parking point along the road network. The length of the shortest path traveled by the vehicle from parking point $p_{k}$ to $p_{k+1}$ is denoted by $d_{k,k+1}$, and the average traveling speed of the vehicle is denoted by $v_{c}$. Accordingly, the corresponding travel time can be expressed as:

(13) $$T_{k,k+1}^{c}=\frac{d_{k,k+1}}{v_{c}}$$

The arrival time of the vehicle at the *k*-th parking point is denoted by $t_{k}^{\mathrm{arr}}$, and the departure time from this parking point is denoted by $t_{k}^{\mathrm{dep}}$. Then, the arrival time at the next parking point satisfies:

(14) $$t_{k+1}^{\mathrm{arr}}=t_{k}^{\mathrm{dep}}+T_{k,k+1}^{c}$$

During the *k*-th parking stage, the vehicle remains stationary at the parking point and is responsible for UAV deployment, waiting during task execution, and unified retrieval. The takeoff time and return time of UAV *i* in the *k*-th parking stage are denoted by $t_{i,k}^{\mathrm{lau}\;}$ and $t_{i,k}^{\mathrm{ret}\;}$, respectively. Under the synchronous cooperation constraint, both its takeoff position and return position should satisfy:

(15) $$r_{i}\left(t_{i,k}^{\mathrm{lau}\;}\right)=p_{k}$$

(16) $$r_{i}\left(t_{i,k}^{\mathrm{ret}\;}\right)=p_{k}$$

The vehicle can leave only after all UAVs involved in the current parking stage have been retrieved. Therefore, the following constraint holds:

(17) $$t_{k}^{\mathrm{dep}\;}\geq t_{i,k}^{\mathrm{ret}\;},\;\forall i\in U$$

By further considering takeoff/landing preparation and retrieval buffer time, the actual vehicle departure time can be written as

(18) $$t_{k}^{\mathrm{dep}\;}=\max_{i\in U}t_{i,k}^{\mathrm{ret}\;}+\Delta t_{k}^{\mathrm{buf}\;}$$

In the equation: $\Delta t_{k}^{\mathrm{buf}\;}$ denotes the buffer time of the *k*-th parking stage.

It can be seen from (18) that whether the task allocation within a single stage is reasonable directly determines the vehicle waiting time at the corresponding parking point. If the task assignment within a stage is excessively concentrated on a small number of UAVs, the vehicle will be forced to wait for the last returning UAV, thereby increasing the total mission completion time of the system. Therefore, under the synchronous cooperative scenario, the task scheduling problem essentially requires a coordinated trade-off among stage partitioning, UAV task allocation, and time overhead.

### 2.4. UAV Dynamics and Task Completion Criterion

To characterize the flight state of UAVs during the inspection process, the velocity vector of UAV *i* at time *t* is defined as:

(19) $$v_{i}(t)={\left[v_{i}^{x}(t),v_{i}^{y}(t),v_{i}^{z}(t)\right]}^{T}$$

Accordingly, its discrete position update equation can be written as:

(20) $$r_{i}(t+\Delta t)=r_{i}(t)+v_{i}(t)\Delta t$$

Considering the flight performance limitations of the UAV, its velocity and attitude variation should satisfy the following constraints:

(21) $$\left\|v_{i}(t)\right\|\leq v_{i}^{\max}$$

(22) $$\left|v_{i}^{z}(t)\right|\leq v_{i,z}^{\max}$$

(23) $$∠\left(v_{i}(t),v_{i}(t-\Delta t)\right)\leq \varphi_{i}^{\max}$$

In the equations: $v_{i}^{\max}$ denotes the maximum flight speed; $v_{i,z}^{\max}$ denotes the maximum vertical speed; and $\varphi_{i}^{\max}$ denotes the maximum allowable turning angle.

The task-point detection radius is defined as $r_{a}$. When the Euclidean distance between UAV *i* and task point *n* satisfies:

(24) $$\left\|r_{i}(t)-q_{n}\right\|\leq r_{a}$$

the *n*-th task point is considered to have been successfully visited by UAV *i*. Accordingly, the task completion state is updated as:

(25) $$y_{n}(t+\Delta t)=\max\left\{y_{n}(t),I\left(\left\|r_{i}(t)-q_{n}\right\|\leq r_{a}\right)\right\}$$

In the equation: $I\left(∙\right)$ denotes the indicator function.

### 2.5. Energy Consumption and State-of-Charge Model

The UAV energy consumption model adopted in this paper is mainly used to describe the basic relationships among flight distance, flight speed, task execution, and SOC evolution under typical inspection conditions, so as to analyze the coupled effects among task allocation, flight energy consumption, and return flight safety margin. It should be noted that this model is a simplified scheduling-level model and does not explicitly consider real operational uncertainties such as strong gust disturbances, temperature variations, payload differences, battery aging, or rotor efficiency changes. Therefore, the SOC results in this paper are mainly intended for relative performance comparison among different scheduling schemes under the same parameter settings, rather than as an absolute prediction of actual UAV endurance in complex field environments.

In the vehicle–UAV cooperative power grid inspection process, UAV energy consumption directly determines the feasible task range and the safety margin for return flight. Since this study focuses on the synchronous cooperative inspection process under normal operating conditions, a simplified energy consumption model without considering extreme wind disturbances is adopted to highlight the fundamental coupling relationship between task scheduling and energy constraints.

The instantaneous power of UAV *i* at time *t* is defined as

(26) $$P_{i}(t)=P_{i}^{\mathrm{hov}\;}+\alpha_{i}{\left\|v_{i}(t)\right\|}^{2}+\beta_{i}\left|v_{i}^{z}(t)\right|$$

In the equation: $P_{i}^{\mathrm{hov}\;}$ denotes the hovering baseline power; $\alpha_{i}$ and $\beta_{i}$ denote the power coefficients for cruising and vertical motion, respectively.

Accordingly, the cumulative energy consumption of UAV *i* over the interval [0, *T*] is given by:

(27) $$E_{i}(T)=\int_{0}^{T}P_{i}(t)dt$$

The maximum battery energy of UAV *i* is denoted by $E_{i}^{\max\;}$, and its remaining energy at time *t* is denoted by $E_{i}^{\mathrm{rem}\;}(t)$. Then, its state of charge (SOC) is defined as:

(28) $$SOC_{i}(t)=\frac{E_{i}^{\mathrm{rem}\;}(t)}{E_{i}^{\max\;}}$$

During flight, the discrete SOC update equation is given by:

(29) $${\mathrm{SOC}}_{i}(t+\Delta t)={\mathrm{SOC}}_{i}(t)-\frac{P_{i}(t)\Delta t}{E_{i}^{\max}}$$

When the UAV returns to the vehicle platform for recharging, the charging power is denoted by $\eta_{i}^{\mathrm{ch}}$, and the SOC is updated as:

(30) $$SOC_{i}(t+\Delta t)=\min\left\{1,SOC_{i}(t)+\frac{\eta_{i}^{\mathrm{ch}}\Delta t}{E_{i}^{\max}}\right\}$$

To ensure task execution safety, the minimum allowable SOC threshold for UAV takeoff is defined as ${\mathrm{SOC}}_{i}^{\mathrm{lau}\;}$. Thus, before executing a new task, the following condition must be satisfied:

(31) $${\mathrm{SOC}}_{i}(t)\geq {\mathrm{SOC}}_{i}^{\mathrm{lau}\;}$$

Furthermore, the minimum residual SOC indicator during the system inspection process is defined as:

(32) $$SOC_{\min}=\min_{i\in U,t\in T}SOC_{i}(t)$$

A larger value of this indicator implies a higher energy safety margin for the system throughout the entire inspection process.

### 2.6. Mission Completion Time and Load Balance Metrics

In multi-UAV cooperative inspection, using task coverage alone as the evaluation criterion is insufficient to comprehensively reflect system performance. Mission completion time can characterize the overall time efficiency, while whether the task allocation among different UAVs is balanced directly affects vehicle waiting time and fleet resource utilization efficiency. Therefore, it is necessary to jointly evaluate the system using the mission completion time and load balance metrics.

The completion time of task point $\tau_{N}$ is denoted by $t_{n}^{\mathrm{fin}}$. Then, the total mission completion time of the system is defined as:

(33) $$T_{\mathrm{fin}}=\max_{1\leq n\leq N}\;t_{n}^{\mathrm{fin}}$$

The number of tasks finally completed by UAV *i* is defined as:

(34) $$N_{i}=\sum_{n=1}^{N}I\left(\tau_{n}\right.\mathrm{finshed}\;\mathrm{by}\;u_{i})$$

Then, the average task load of all UAVs is:

(35) $$\bar{N}=\frac{1}{K}\sum_{i=1}^{K}N_{i}$$

The load balance metric of the UAV fleet is defined as:

(36) $$B=\frac{1}{K}\sum_{i=1}^{K}{\left(N_{i}-\bar{N}\right)}^{2}$$

A smaller value of *B* indicates that the task loads among UAVs are more balanced and that the cooperative task allocation is more reasonable.

### 2.7. Multi-Objective Task Scheduling Model

By comprehensively considering inspection completeness, mission time efficiency, energy safety, and fleet coordination, the vehicle–UAV synchronous cooperative power grid inspection scheduling problem is formulated in this paper as the following multi-objective optimization model:

(37) $$\max\rho \left(T_{\mathrm{fin}}\right)$$

(38) $$\min T_{\mathrm{fin}}$$

(39) $$\max SOC_{\min}$$

(40) minB

To facilitate the subsequent unified solution by NSGA-II, the above objectives are transformed into minimization form, yielding the objective vector:

(41) $$F(\theta )=\left[1-\rho \left(T_{\mathrm{fin}}\right),T_{\mathrm{fin}},-SOC_{\min},B\right]$$

In the equation: *θ* denotes the scheduling parameter vector to be optimized.

## 3. Multi-Objective Optimization Method Based on MOTD3–NSGA-II

### 3.1. Overall Methodology

**Figure 1:**
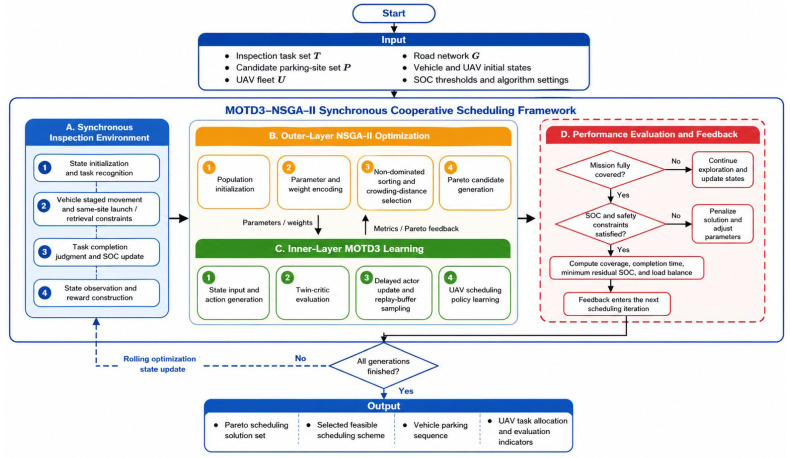
Framework of the MOTD3–NSGA-II bi-level optimization method.

For the vehicle–UAV synchronous cooperative power grid inspection task scheduling model established in Section 2, this paper proposes a bi-level optimization method based on MOTD3–NSGA-II. Since the vehicle–UAV cooperative inspection process simultaneously involves high-dimensional continuous-action decision-making and multi-objective parameter trade-offs, directly applying a single-layer solution method may lead to an excessively large search space, unstable policy training, and insufficient multi-objective coordination capability. Therefore, a hierarchical solution framework of “outer-layer parameter optimization and inner-layer policy learning” is adopted. Specifically, the outer-layer NSGA-II is responsible for searching system-level cooperative parameters and reward preference weights, while the inner-layer MOTD3 learns the scheduling and control policy for multiple UAVs in a continuous action space under given parameter settings, thereby achieving comprehensive optimization of task coverage, mission completion time, minimum SOC, and load balance. The specific flowchart is shown in Figure 1.

The core idea of the proposed method is to decompose the vehicle–UAV synchronous cooperative problem, which is difficult to solve in a unified manner, into two interrelated subproblems: global parameter optimization and local policy learning. The former focuses on macroscopic parameters such as stage partitioning scale, arrival detection radius, takeoff SOC threshold, velocity scaling factor, and reward preference weights. The latter focuses on operational decision-making during task execution, including target selection, velocity adjustment, return decision-making, and cooperative control among UAVs. Through the closed-loop interaction of the bi-level structure, the proposed method enhances the stability and practicality of the reinforcement learning policy in complex scheduling environments while preserving multi-objective optimization capability.

The overall pseudocode is presented in Algorithm 1.
**Algorithm 1.** Bi-level MOTD3–NSGA-II for vehicle–UAV synchronous cooperative inspection scheduling**Input:** Inspection task set T, candidate parking-site set P, UAV fleet U, road network G, parameter search space Θ$,\;\mathrm{maximum}\;\mathrm{NSGA}\text{-}\mathrm{II}\;\mathrm{generations}\;G_{max}$$,\;\mathrm{population}\;\mathrm{size}\;N_{p}$$,\;\mathrm{maximum}\;\mathrm{training}\;\mathrm{episodes}\;E_{max}$, and maximum decision horizon H. **Output:** $\mathrm{Pareto}\;\mathrm{scheduling}\;\mathrm{solution}\;\mathrm{set}\;S_{Pareto}$ and selected feasible scheduling scheme θ*. 1:   Initialize the NSGA-II population within the parameter search space Θ 2:   Initialize the Pareto solution archive S_Pareto 3:   for generation = 1 to Gmax do 4:    for each individual θ in the current population do 5:     Construct the synchronous vehicle–UAV inspection environment under θ 6:     Initialize the actor network, twin critic networks, target networks, and replay buffer of MOTD3 7:     for episode = 1 to Emax do 8:      Reset the inspection environment and obtain the initial state 9:      for t = 1 to H do 10:        Generate continuous UAV scheduling actions using the actor network 11:        Execute the actions in the synchronous inspection environment 12:        Observe the next state, multi-component reward, and termination flag 13:        Compute the scalar reward according to the preference weights 14:        Store the transition sample in the replay buffer 15:        Update the twin critic networks using sampled mini-batches 16:        Update the actor and target networks according to the delayed policy update rule 17:        if the termination condition is satisfied then 18:         break 19:        end if 20:       end for 21:      end for 22:      Evaluate the current individual using task coverage, mission completion time,        minimum residual SOC, and load balance 23:     end for 24:     Perform non-dominated sorting and crowding-distance calculation 25:     Select parent individuals and generate offspring through crossover and mutation 26:     Update the NSGA-II population and the Pareto solution archive 27: end for 28: Select the final feasible scheduling scheme from S_Pareto according to   inspection completeness, SOC safety, and mission completion time 29: return S_Pareto and θ*

### 3.2. Outer-Layer Parameter Optimization Model

In the outer-layer optimization, the key parameters affecting the scheduling performance of vehicle–UAV synchronous cooperation are combined into a parameter vector:

(42) $$\theta =\left[r_{a},r_{\mathrm{rec}},SOC_{\min}^{\mathrm{lau}},\eta^{\mathrm{ch}},\kappa_{v},w_{1},w_{2},w_{3},w_{4}\right]$$

In the equation: $r_{a}$ denotes the task-point arrival detection radius; $r_{\mathrm{rec}}$ denotes the retrieval detection radius; $SOC_{\min}^{\mathrm{lau}}$ denotes the minimum takeoff SOC threshold; $\eta^{\mathrm{ch}}$ denotes the charging power; $\kappa_{v}$ denotes the velocity scaling factor; $w_{1}$ to $w_{4}$ denote the preference weights of each objective component in the inner-layer reward function.

For any parameter combination *θ* given by the outer layer, the inner-layer MOTD3 trains or executes the corresponding policy in the associated environment and outputs the resulting inspection performance. Thus, the mapping from the parameter vector to the objective function can be expressed as:

(43) $$F(\theta )=\mathrm{Eval}\left(\pi_{\mathrm{MOTD}3}^{*}(;\theta )\right)$$

In the equation: $\pi_{\mathrm{MOTD}3}^{*}$ denotes the optimal policy obtained under parameter *θ*; $\mathrm{Eval}\left(∙\right)$ denotes the evaluation of the policy in terms of task coverage, mission completion time, minimum SOC and load balance.

The outer-layer NSGA-II searches the parameter vector based on a population evolution mechanism and obtains the Pareto solution set through fast nondominated sorting. Let the ranking position of the *m*-th normalized objective function on the same nondominated front be *j*. Then, the crowding distance of individual *i* can be written as:

(44) $$D_{i}=\sum_{m=1}^{4}\frac{f_{m}^{\left(j+1\right)}-f_{m}^{\left(j-1\right)}}{f_{m}^{\max}-f_{m}^{\min}}$$

In the equation: $f_{m}^{\left(j+1\right)}$ and $f_{m}^{\left(j-1\right)}$ denote the function values of the two neighboring individuals on the *m*-th objective, respectively. A larger crowding distance indicates that the individual lies in a sparser region and should therefore be assigned a higher priority for preservation during environmental selection. Through the joint screening of nondominated rank and crowding distance, NSGA-II can maintain the diversity of the solution set while ensuring convergence, thereby providing multiple candidate solutions for subsequent decision-making.

### 3.3. Inner-Layer Cooperative Decision-Making Model

In the inner-layer optimization, the vehicle–UAV synchronous cooperative inspection process is modeled as a multi-agent Markov decision process in a continuous action space. The system state includes not only the current vehicle parking stage, UAV positions and velocities, remaining battery energy, and the distribution of completed tasks, but also the remaining tasks to be executed in the current stage and the vehicle waiting status. In this way, the policy network can perceive the cooperative scheduling environment from a global perspective.

The system state at time *t* is denoted by *s_t_*, and the continuous action output of UAV *i* is denoted by **a**_i,t_. Then, the joint action can be written as:

(45) $$a_{t}=\left[a_{1,t},a_{2,t},\cdots ,a_{K,t}\right]$$

To accommodate the multi-objective optimization requirement, the instantaneous reward is designed in a multi-component form as:

(46) $$r_{t}={\left[\Delta \rho_{t},-\Delta t,-\Delta e_{t},-\Delta b_{t}\right]}^{T}$$

In the equation: $\Delta \rho_{t}$ denotes the increment in task coverage per unit time; Δt denotes the time advancement cost; $\Delta e_{t}$ denotes the increment in energy consumption; $\Delta b_{t}$ denotes the variation in load imbalance.

By further incorporating the preference weight vector $w={\left[w_{1},w_{2},w_{3},w_{4}\right]}^{T}$ given by the outer layer, the scalar reward function is constructed as:

(47) $$r_{t}=w^{T}r_{t}$$

This design allows the outer-layer optimization to adjust the preference direction of the inner-layer policy by tuning the weight vector, thereby establishing a configurable coordination mechanism among different performance objectives.

### 3.4. MOTD3 Policy Learning Mechanism

MOTD3 introduces the multi-objective task scheduling scenario into the TD3 framework. By employing the twin-Critic structure, delayed policy updates, and target policy smoothing, it alleviates the common problems of action-value overestimation and training oscillation in continuous-action reinforcement learning.

The target policy network is denoted by $\pi_{\bar{\omega }}$. Then, by adding smoothing noise during target action computation, one obtains:

(48) $${\tilde{a}}_{t+1}=\pi_{\bar{\omega }}\left(s_{t+1}\right)+\mathrm{clip}\left(\xi_{t+1},-\delta ,\delta \right),\;\xi_{t+1}\sim N\left(0,\sigma_{\xi }^{2}\right)$$

In the equation: $\sigma_{\xi }$ denotes the standard deviation of the noise; δ denotes the clipping threshold.

Under the twin-Critic structure, the target Q-value is defined as:

(49) $$y_{t}=r_{t}+\gamma \min\left\{Q_{{\bar{\psi }}_{1}}\left(s_{t+1},{\tilde{a}}_{t+1}\right),Q_{{\bar{\psi }}_{2}}\left(s_{t+1},{\tilde{a}}_{t+1}\right)\right\}$$

In the equation: *γ* denotes the discount factor; $Q_{{\bar{\psi }}_{1}}$ and $Q_{{\bar{\psi }}_{2}}$ denote the two target Critic networks.

Correspondingly, the loss function of the *m*-th Critic network is written as:

(50) $$L\left(\psi_{m}\right)=\frac{1}{N_{b}}\sum_{l=1}^{N_{b}}{\left[Q_{\psi_{m}}\left(s^{\left(l\right)},a^{\left(l\right)}\right)-y^{\left(l\right)}\right]}^{2},m\in \{1,2\}$$

In the equation: *N_b_* denotes the mini-batch size; $\left(s^{\left(l\right)},a^{\left(l\right)},y^{\left(l\right)}\right)$ denotes the l-th sampled transition.

To further improve training stability, MOTD3 does not update the Actor network at every step, but instead adopts a delayed update strategy. Suppose that the Actor is updated once after every *N_u_* Critic updates. Then, the policy gradient of the Actor can be expressed as:

(51) $$\nabla_{\omega }J(\omega )={\left.\frac{1}{N_{b}}\sum_{l=1}^{N_{b}}\nabla_{a}Q_{\psi_{1}}\left(s^{\left(l\right)},a\right)\right|}_{a=\pi_{\omega }\left(s^{\left(l\right)}\right)}\cdot \nabla_{\omega }\pi_{\omega }\left(s^{\left(l\right)}\right)$$

Meanwhile, to improve training stability, the target networks are updated using soft updates:

(52) $${\bar{\psi }}_{m}\leftarrow \varrho \psi_{m}+(1-\varrho ){\bar{\psi }}_{m},\;m\in \{1,2\}$$

(53) $$\bar{\omega }\leftarrow \varrho \omega +(1-\varrho )\bar{\omega }$$

In the equation: ϱ∈(0,1) denotes the soft update coefficient.

Through the above mechanisms, MOTD3 can stably learn scheduling and control policies suitable for vehicle–UAV synchronous cooperative inspection scenarios in complex continuous action spaces, thereby effectively improving the convergence and robustness of policy training.

### 3.5. Closed-Loop Interaction Mechanism and Final Solution Selection

In the bi-level framework proposed in this paper, NSGA-II and MOTD3 are not independent of each other, but are coupled through a closed-loop procedure of “parameter generation–policy learning–performance feedback.” Specifically, the outer-layer NSGA-II first generates a candidate parameter vector *θ*. Then, under the corresponding parameter settings, the inner-layer MOTD3 interacts with the simulation environment to produce UAV scheduling policies and inspection results. Subsequently, the evaluation module computes the task coverage, mission completion time, minimum residual SOC, and load balance metric, and feeds them back to NSGA-II as the fitness of the outer-layer population individual. After multiple iterations, the system obtains a set of Pareto-optimal solutions with different performance emphases.

For subsequent description, the final task coverage is defined as:

(54) $$\rho_{\mathrm{fin}}=\rho \left(T_{\mathrm{fin}}\right)$$

Considering that practical engineering applications usually require a unique scheduling solution, this paper further adopts a final solution selection strategy of “inspection completeness and safety first, followed by efficiency.” Let the Pareto solution set be $\Omega_{P}$, and the feasible solution set satisfying the requirements of full coverage and energy safety is extracted as:

(55) $$\Omega_{F}=\left\{\theta \in \Omega_{P}|\rho_{\mathrm{fin}}=1,SOC_{\min}\geq SOC_{\mathrm{safe}}\right\}$$

In the equation: *SOC*_safe_ denotes the minimum safe state-of-charge threshold of the system.

If $\Omega_{F}\neq \emptyset$, the solution with the shortest mission completion time is selected as the final scheduling solution:

(56) $$\theta^{*}=\arg\min_{\theta \in \Omega_{F}}T_{\mathrm{fin}}$$

If the feasible solution set is empty in certain complex scenarios, a degraded screening strategy is adopted according to the priority order of “coverage first, energy safety second, and mission completion time third,” so as to ensure that the output solution still maintains satisfactory engineering applicability.

## 4. Case Studies and Analysis

### 4.1. Simulation Scenario Construction and Task Region Partitioning

To verify the applicability of the proposed vehicle–UAV synchronous cooperative inspection method under different task distribution conditions, four representative simulation scenarios for distribution network inspection are constructed in this paper. Each scenario includes a road network, a set of parking points, and discrete task points distributed along both sides of the roads and near the regional boundaries. The vehicle performs stage-wise repositioning along the predetermined road network, while the UAVs complete takeoff, inspection, and retrieval at the corresponding parking points. The scenario parameters of the four scenarios are listed in Table 1.

**Figure 2:**
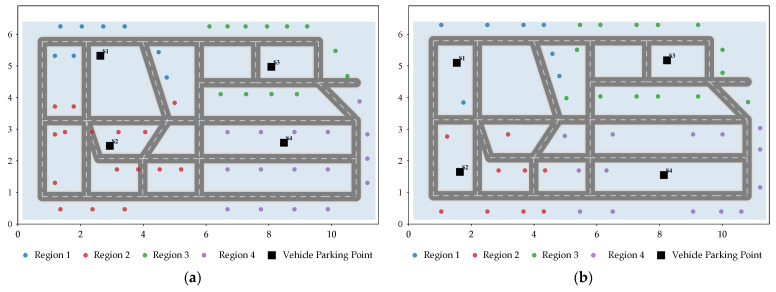

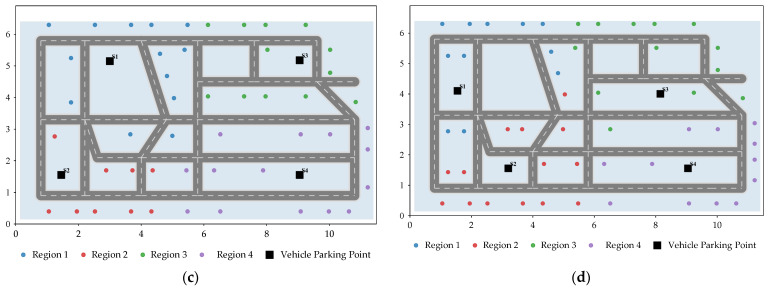
Diagram of different scenarios and task allocation. (**a**) Scenario 1; (**b**) Scenario 2; (**c**) Scenario 3; (**d**) Scenario 4.

After constructing the basic scenarios, task points in each scenario are further partitioned into different regions according to the locations of the parking points and the spatial distribution of the tasks, as shown in Figure 2. Different colors in the figure represent the task regions primarily served by the four parking points, and the black squares denote the parking-point locations. Overall, the partitioning results show high consistency with the spatial distribution of the parking points. Task points located in relatively independent areas, such as the upper-left, lower-left, upper-right, and lower-right regions, are assigned to the parking points that are spatially closer and more convenient for service. For task points near road intersections or regional boundaries, the partitioning results also take into account both spatial proximity and stage execution convenience, thereby reducing the additional overhead caused by cross-regional execution and repeated scheduling under the synchronous cooperative mode. These results indicate that task region partitioning not only provides a clear spatial organizational basis for subsequent vehicle–UAV synchronous cooperative scheduling, but also helps reduce vehicle waiting time and improve the rationality of UAV task allocation.

### 4.2. Analysis of the Vehicle–UAV Synchronous Cooperative Execution Process Under Different Scenarios

**Figure 3:**
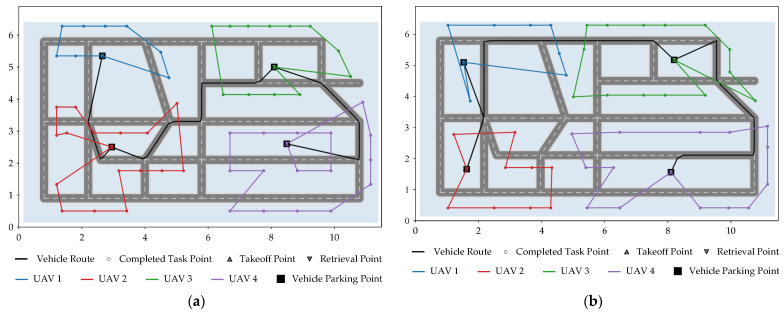

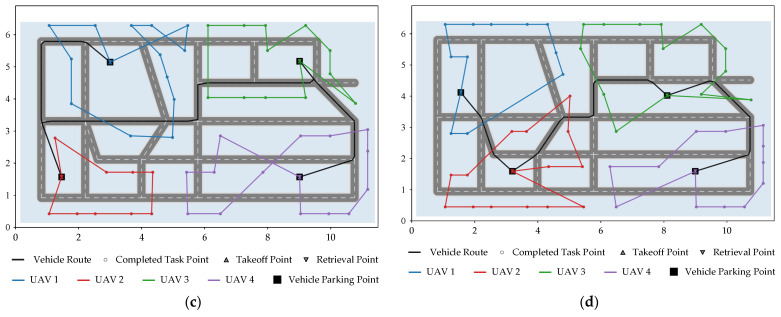
Planar cooperative trajectories in different scenarios. (**a**) Scenario 1; (**b**) Scenario 2; (**c**) Scenario 3; (**d**) Scenario 4.

**Figure 4:**
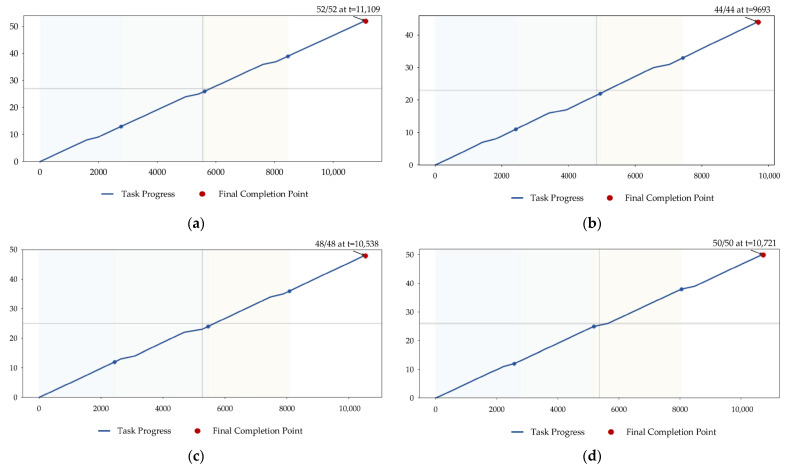
Task completion progress curves in different scenarios. (**a**) Scenario 1; (**b**) Scenario 2; (**c**) Scenario 3; (**d**) Scenario 4.

**Figure 5:**
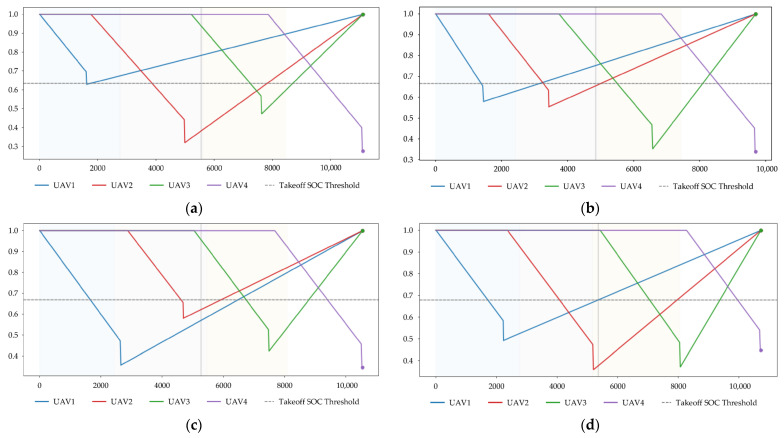
SOC evolution of the UAV fleet in different scenarios. (**a**) Scenario 1; (**b**) Scenario 2; (**c**) Scenario 3; (**d**) Scenario 4.

**Figure 6:**
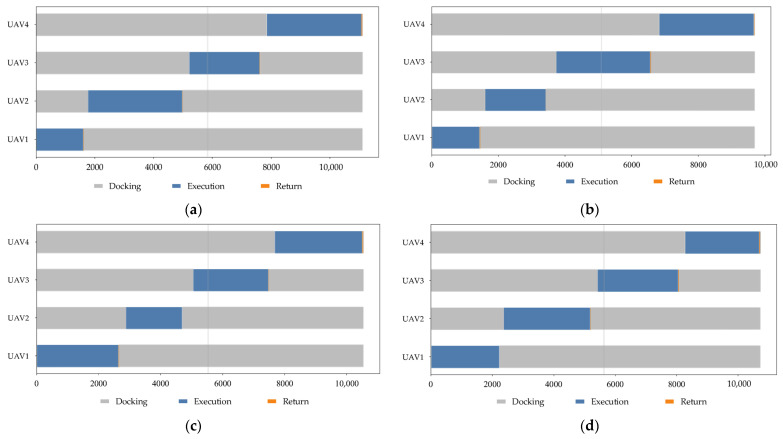
UAV state timeline diagrams in different scenarios. (**a**) Scenario 1; (**b**) Scenario 2; (**c**) Scenario 3; (**d**) Scenario 4.

After completing the scenario construction and task region partitioning, the execution process of vehicle–UAV synchronous cooperative inspection in the four simulation scenarios is further analyzed. To ensure consistency in comparison, the same cooperative framework is adopted in all four scenarios. Specifically, the vehicle performs stage-wise repositioning among the four parking points along the predetermined road network, while the UAVs take off from the corresponding parking point during each vehicle parking stage, execute the assigned tasks, and return to the same parking point for retrieval and recharging after completing the tasks of the current stage. The planar cooperative trajectories, task completion progress, UAV fleet SOC evolution, and state time-series results for the four scenarios are shown in Figure 3, Figure 4, Figure 5 and Figure 6, respectively.

As can be seen in Figure 3, in all four scenarios, the vehicle strictly completes the ordered repositioning among parking points along the road network, whereas the four UAVs perform inspection tasks around their respective assigned task regions, thereby forming a relatively clear regional division of labor. Tasks located in the upper-left, lower-left, upper-right, and lower-right areas are generally completed first by UAVs supported by the neighboring parking points. No obvious large-scale cross-regional flights or repeated revisits are observed, indicating that the stage-wise execution strategy based on task region partitioning can effectively reduce the disorder of scheduling under the synchronous cooperative mode.

From Figure 4, it can be observed that the completion curves in all four scenarios increase monotonically over time and eventually achieve full-coverage inspection. Specifically, Scenarios 1–4 complete all tasks at *t* = 11,109 s, *t* = 9693 s, *t* = 10,538 s and *t* = 10,721 s, respectively, corresponding to 52, 44, 48, and 50 task points. It can be seen that the task advancement process in different scenarios exhibits evident stage-wise characteristics. During stages in which tasks are relatively concentrated and route connections are smoother, the task completion rate is relatively high; whereas in the terminal phase of a stage or during the clearance of boundary tasks, the progression slope decreases to some extent. This result is consistent with the operational mechanism of vehicle–UAV synchronous cooperative inspection, in which the vehicle can move to the next parking point only after all tasks in the current stage have been completed. Therefore, both task scale and spatial distribution characteristics directly affect the convergence speed at the end of each stage.

From Figure 5, it can be observed that all UAVs in the four scenarios complete the full cycle of “task execution–return–recharging,” with no task interruption or abnormal hovering caused by insufficient energy. According to the curves, the minimum residual SOC values in Scenarios 1–4 are approximately 0.28, 0.34, 0.35 and 0.36, respectively. In general, scenarios with larger task scales and longer local task paths tend to exhibit lower minimum residual SOC, whereas scenarios with more balanced task distributions or moderately fewer tasks provide a better overall energy margin.

Meanwhile, the switching sequence among the “docking–execution–return” states is clear in Figure 6, and the stage boundaries are well defined, indicating that the proposed method can stably maintain the execution logic of synchronous cooperative inspection under different task scales and spatial distribution conditions.

In summary, Figure 3, Figure 4, Figure 5 and Figure 6 show that the proposed method can effectively coordinate vehicle repositioning, UAV task allocation, task advancement, and energy management across all four scenarios, thereby demonstrating good execution completeness and scenario adaptability.

### 4.3. Statistical Results and Adaptability Analysis Across Multiple Scenarios

To further verify the applicability of the proposed method under different task scales and spatial distribution conditions, the overall results of the four simulation scenarios are summarized in a unified manner, as listed in Table 2. It can be seen that the proposed vehicle–UAV synchronous cooperative inspection method achieves full-coverage inspection with a task coverage of 1.000 in all four scenarios, indicating that the method has stable task completion capability.

In terms of mission completion time, the total completion times of the four scenarios range from *t* = 9693 s to *t* = 11,109 s. Among them, Scenario 2 contains the fewest task points and therefore achieves the shortest completion time, whereas Scenario 1 has the largest task scale and correspondingly the longest completion time. Scenarios 3 and 4 fall between these two cases. These results indicate that the total mission completion time of synchronous cooperative inspection is closely related to both task scale and spatial distribution characteristics. When the number of tasks increases or more boundary tasks are involved, the stage-finishing time of the system generally increases, thereby prolonging the overall execution duration.

In terms of energy safety, the minimum residual SOC in the four scenarios ranges from 0.28 to 0.36, remaining overall within an acceptable range. Although some differences exist among the scenarios, no return failure or execution interruption caused by excessive energy consumption of a single UAV is observed in any scenario, indicating that the vehicle-based recharging mechanism and the UAV stage-wise execution strategy can effectively support multi-scenario inspection tasks. Overall, the proposed method demonstrates good full-coverage capability, a stable task advancement process, and an acceptable energy margin in all four scenarios, thereby verifying its feasibility and applicability in multi-scenario vehicle–UAV synchronous cooperative power grid inspection tasks.

### 4.4. Comparative Analysis of Different Scheduling Methods and Pareto-Set Quality

To further verify the effectiveness of the proposed MOTD3–NSGA-II bi-level optimization framework and strengthen the horizontal comparison among different scheduling methods, this section introduces two additional representative multi-objective optimization baselines, namely NSGA-III and MOEA/D, in addition to Greedy heuristic, NSGA-II-only, and MOTD3-only. NSGA-III improves the distribution of solutions in relatively high-dimensional objective spaces through a reference-point mechanism, whereas MOEA/D decomposes a multi-objective optimization problem into a set of scalar subproblems and solves them collaboratively. In contrast, the proposed MOTD3–NSGA-II method uses the outer-layer NSGA-II to search cooperative parameters and objective preference weights, while the inner-layer MOTD3 learns UAV scheduling policies in a continuous action space, thereby integrating multi-objective trade-off capability with continuous policy learning.

The compared methods in this section include Greedy heuristic, NSGA-II-only, MOTD3-only, NSGA-III, MOEA/D, and MOTD3–NSGA-II. The Greedy heuristic is used to represent a simple heuristic task allocation strategy. NSGA-II-only represents a scheduling method that retains only the outer-layer multi-objective evolutionary search without introducing inner-layer policy learning. MOTD3-only represents a single reinforcement learning method with fixed objective preference weights. NSGA-III and MOEA/D are used as representative multi-objective evolutionary optimization baselines. MOTD3–NSGA-II denotes the proposed method. All methods are evaluated using the same task-point set, vehicle road network, parking-site sequence, number of UAVs, initial SOC, safety threshold, and task completion criterion, so that the comparison mainly reflects the differences among the scheduling methods themselves.

Table 3 presents the comparison results of different scheduling methods under multiple evaluation indicators. The evaluation indicators include task coverage, mission completion time, minimum residual SOC, and load balance index. Among them, task coverage and minimum residual SOC are benefit-type indicators, for which larger values indicate better performance. Mission completion time and load balance index are cost-type indicators, for which smaller values indicate better performance.

As shown in Table 3, although the Greedy heuristic is easy to implement, it mainly relies on local task selection and lacks global multi-objective trade-off capability, resulting in relatively weak performance in task coverage, mission completion time, energy margin, and load balance. The NSGA-II-only method performs better than the Greedy heuristic, indicating that multi-objective evolutionary search can provide a certain degree of coordination among task coverage, time efficiency, and energy safety. However, because this method lacks a continuous-action policy learning mechanism, its fine-grained scheduling capability during the execution process remains limited. The MOTD3-only method can learn scheduling policies based on environmental states, and its task coverage and mission completion time are better than those of the first two methods. Nevertheless, the fixed objective preference weights restrict its ability to flexibly balance multiple objectives.

A further comparison with NSGA-III and MOEA/D shows that these two competitive multi-objective optimization methods outperform Greedy heuristic, NSGA-II-only, and MOTD3-only in terms of task coverage, mission completion time, and minimum residual SOC. This indicates that stronger multi-objective search mechanisms can improve cooperative scheduling performance. However, these two methods mainly focus on outer-layer multi-objective search and still have limited capability in modeling continuous-action feedback and within-stage dynamic scheduling during UAV execution. By contrast, MOTD3–NSGA-II achieves the best results in task coverage, mission completion time, minimum residual SOC, and load balance index, indicating that outer-layer multi-objective search and inner-layer continuous policy learning can complement each other.

**Figure 7:**
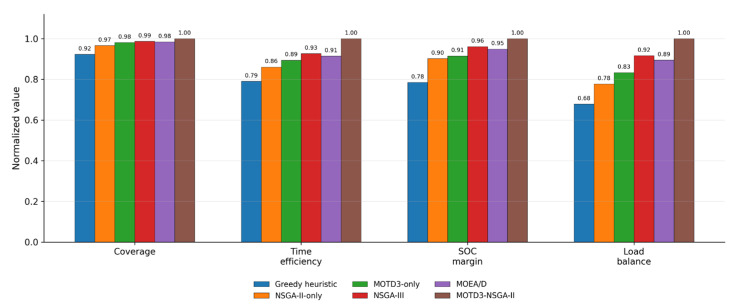
Normalized comparison of different scheduling methods under multiple evaluation indicators.

Figure 7 further provides an intuitive comparison of different scheduling methods under normalized indicators. It can be seen that MOTD3–NSGA-II achieves the best overall performance in task coverage, time efficiency, SOC margin, and load balance. NSGA-III and MOEA/D perform better than the simple heuristic and single-module methods, but remain inferior to the proposed method. This indicates that the advantage of the proposed method does not come from a single evolutionary search process or a single reinforcement learning structure alone, but from the closed-loop cooperation between outer-layer multi-objective parameter search and inner-layer continuous-action policy learning.

To further evaluate the quality of the Pareto solution sets obtained by different multi-objective methods, this paper introduces three standard multi-objective performance metrics, namely Hypervolume (HV), Inverted Generational Distance (IGD), and Spacing. HV is used to comprehensively evaluate the convergence and coverage of the obtained solution set; a larger HV value indicates better Pareto-set quality. IGD measures the distance between the obtained solution set and the reference Pareto front; a smaller IGD value indicates better convergence. Spacing evaluates the distribution uniformity of the obtained solutions; a smaller Spacing value indicates a more uniform distribution.

As shown in Table 4, MOTD3–NSGA-II obtains the largest number of nondominated solutions, the highest HV value, and the lowest IGD and Spacing values. This demonstrates that the proposed method outperforms the comparison methods in terms of Pareto-set convergence, coverage, and distribution uniformity. NSGA-III achieves relatively good performance in solution distribution owing to its reference-point mechanism, and MOEA/D can also obtain stable multi-objective solutions through its decomposition mechanism. However, because both methods lack the inner-layer continuous-action policy learning mechanism, their final scheduling performance and Pareto-set quality are still inferior to those of the proposed method.

**Figure 8:**
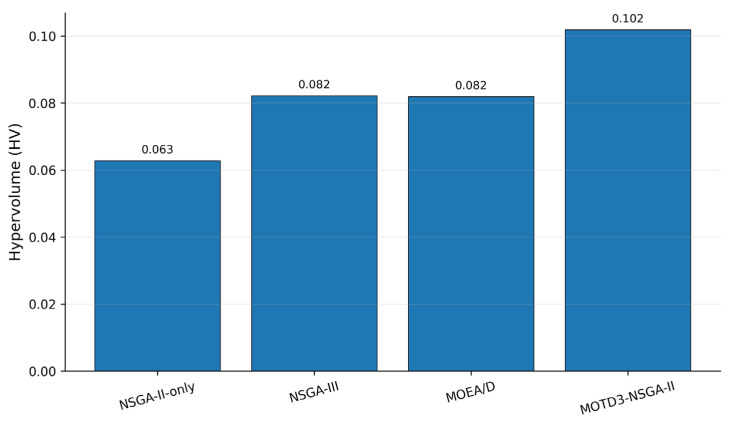
HV comparison of different multi-objective methods.

**Figure 9:**
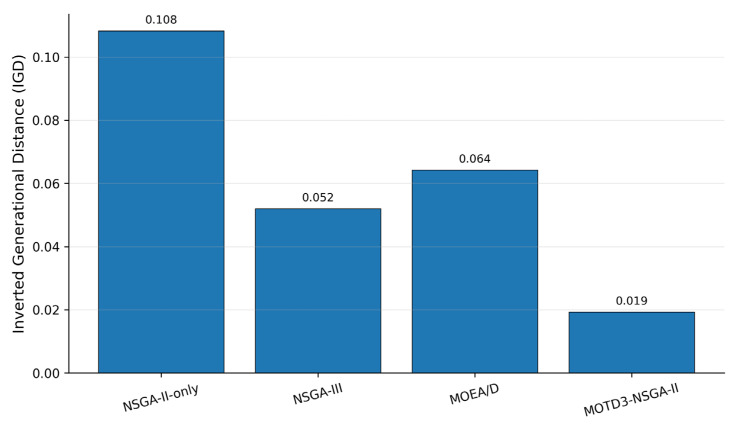
IGD comparison of different multi-objective methods.

Figure 8 and Figure 9 show the HV and IGD comparison results of different multi-objective methods, respectively. MOTD3–NSGA-II achieves a clearly higher HV value than NSGA-II-only, NSGA-III, and MOEA/D, while also obtaining the lowest IGD value. These results indicate that the proposed method not only outputs a high-performance scheduling scheme, but also obtains a Pareto solution set that is closer to the reference front and better distributed in the multi-objective sense. This further complements the evaluation based solely on normalized single-solution indicators.

**Figure 10:**
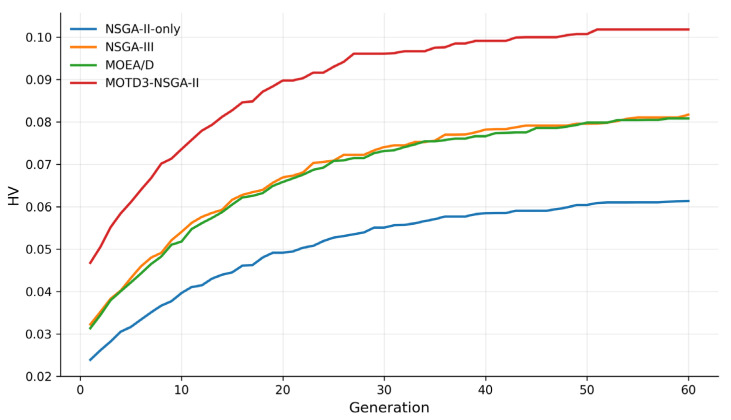
HV convergence curves of different methods.

Figure 10 shows the HV convergence process of different multi-objective methods. It can be observed that the HV values of all methods increase gradually with the number of generations and eventually tend to stabilize. Among them, MOTD3–NSGA-II maintains a higher HV level throughout the iteration process and reaches the highest converged value in the later stage. This indicates that the proposed bi-level framework has good convergence performance under the current problem scale.

It should be noted that the problem studied in this paper contains four objectives, namely task coverage, mission completion time, minimum residual SOC, and load balance, and therefore belongs to a relatively high-dimensional multi-objective optimization problem. As the number of objectives increases, NSGA-II may suffer from reduced selection pressure in nondominated sorting. The main reasons for adopting NSGA-II in this study are as follows. First, the four objectives considered in this problem are not completely independent; mission completion time, SOC margin, and load balance exhibit certain coupling relationships. Second, in the proposed bi-level framework, NSGA-II mainly serves as the outer-layer search mechanism for cooperative parameters and objective preference weights, while the inner-layer MOTD3 is responsible for learning continuous-action scheduling policies during task execution. The comparison with NSGA-III and MOEA/D shows that, under the current problem scale and synchronous cooperation constraints, the combination of NSGA-II and inner-layer MOTD3 can still achieve good Pareto-set quality and scheduling performance.

In addition, the inner-layer MOTD3 adopts a linear weighted form to transform the multi-component reward into a scalar reward. This design facilitates the embedding of multi-objective preferences into the continuous-action reinforcement learning framework. However, linear scalarization has limited capability in representing non-convex Pareto fronts. In this study, the outer-layer NSGA-II searches different preference weight combinations, which alleviates the limitation of a single fixed preference to some extent. Nevertheless, this strategy is still not equivalent to a strict Pareto-based reinforcement learning method. Future work will further consider preference-conditioned policies, Pareto-based multi-objective reinforcement learning, or constrained reinforcement learning to better characterize non-convex Pareto fronts and more complex multi-objective preferences.

### 4.5. Robustness Under Uncertainty and Scalability Analysis

To further examine the stability of the proposed method under conditions closer to real-world operation, this section introduces stochastic perturbations into the deterministic simulation setting and conducts robustness experiments under uncertainty. Meanwhile, by varying the task scale, number of UAVs, and environment size, the runtime and scalability of the proposed method are further analyzed.

#### 4.5.1. Robustness Analysis Under Stochastic Perturbations

In practical distribution network inspection, vehicle transfer time, UAV energy consumption, and task execution are often affected by road traffic conditions, local wind disturbances, payload variations, sensing errors, and on-site execution delays. To enhance the realism of the experimental evaluation, three types of stochastic perturbations are introduced into the simulation: vehicle transfer time perturbation, UAV energy consumption perturbation, and task execution/sensing perturbation. Specifically, vehicle transfer time is randomly varied around its nominal value, UAV energy consumption parameters are perturbed within a given range, and small random errors are introduced into the task completion judgment process. All methods are repeatedly tested under the same perturbation conditions, and task coverage, mission completion time, minimum residual SOC, load balance index, and full-mission success rate are recorded.

Task coverage reflects the average inspection completion capability of each method under perturbations. The full-mission success rate indicates the proportion of trials in which both the task coverage requirement and the SOC safety constraint are satisfied under stochastic perturbations. Unlike deterministic simulations, stochastic perturbations may affect vehicle arrival time, UAV return flight margin, and the final stage-finishing process. Therefore, the corresponding indicators are reported in the form of mean ± standard deviation.

As shown in Table 5, under stochastic perturbations, the task coverage, mission completion time, and minimum residual SOC of different methods all fluctuate to varying degrees. Since the Greedy heuristic mainly relies on local nearest-task selection and lacks global coordination capability for stage waiting and energy margins under perturbations, its task coverage and full-mission success rate are relatively low. The NSGA-II-only method performs better than the Greedy heuristic, indicating that multi-objective evolutionary search can improve the coordination between task allocation and energy constraints. However, owing to the absence of an inner-layer continuous-action policy learning mechanism, its adaptability during execution under stochastic perturbations remains limited.

The MOTD3-only method can learn continuous-action policies according to environmental states, thereby improving task coverage and full-mission success rate under perturbations. However, because this method uses fixed objective preference weights, it is difficult to flexibly balance task coverage, mission completion time, SOC margin, and load balance. Therefore, its overall performance is still weaker than those of the multi-objective optimization baselines and the proposed method. NSGA-III and MOEA/D also show good robustness under perturbations, indicating that the reference-point mechanism and decomposition mechanism can improve the stability of multi-objective scheduling schemes to some extent. In contrast, MOTD3–NSGA-II maintains 100.00% task coverage and 100.00% full-mission success rate under stochastic perturbations, while also achieving the best mission completion time, minimum residual SOC, and load balance index. These results demonstrate that the proposed method has better overall robustness under moderate perturbation conditions.

**Figure 11:**
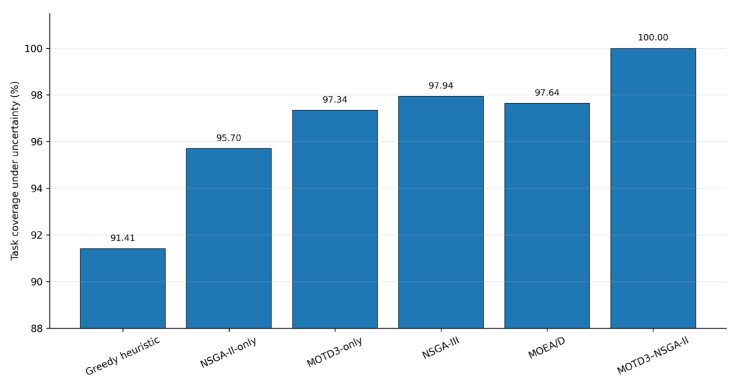
Task coverage comparison of different methods under stochastic perturbations.

Figure 11 compares the task coverage of different scheduling methods under stochastic perturbations. It can be seen that when vehicle transfer time, UAV energy consumption, and task completion judgment are all subject to random disturbances, the task coverage of the simple heuristic and single-layer optimization methods decreases more evidently. In contrast, MOTD3-only, NSGA-III, and MOEA/D show stronger disturbance resistance. MOTD3–NSGA-II achieves an average task coverage of 100.00%, which is the highest among all methods, and its standard deviation is 0.00, indicating that the proposed method can maintain stable task completion capability under the considered perturbation settings.

#### 4.5.2. Computational Efficiency and Scalability Analysis

In addition to robustness, computational efficiency and scalability are also important for vehicle–UAV cooperative inspection scheduling methods. To analyze the performance of the proposed method under different problem scales, four types of scenarios, namely Small, Reference, Medium, and Large, are constructed by gradually increasing the number of task points, the number of UAVs, and the environment size. The runtime, task coverage, mission completion time, and minimum residual SOC are then recorded.

As shown in Table 6, as the number of task points and environment size increase, the runtime increases from 31.8 s in the Small scenario to 124.5 s in the Large scenario, and the mission completion time also increases with the task scale. This trend is consistent with the basic characteristics of vehicle–UAV cooperative inspection tasks, where more task points and a larger spatial range increase the complexity of vehicle transfer, UAV flight, and task allocation. In terms of task coverage, the Small and Reference scenarios both achieve 100.00% coverage, while the Medium and Large scenarios achieve task coverage values of 99.10% and 98.40%, respectively. This indicates that the proposed method can still maintain high task completion capability when the task scale increases significantly. In terms of energy safety, the minimum residual SOC decreases as the task scale increases, but it remains 25.60% even in the Large scenario, indicating that the vehicle-based recharging and stage-wise scheduling mechanism can maintain a certain energy safety margin in larger-scale scenarios.

**Figure 12:**
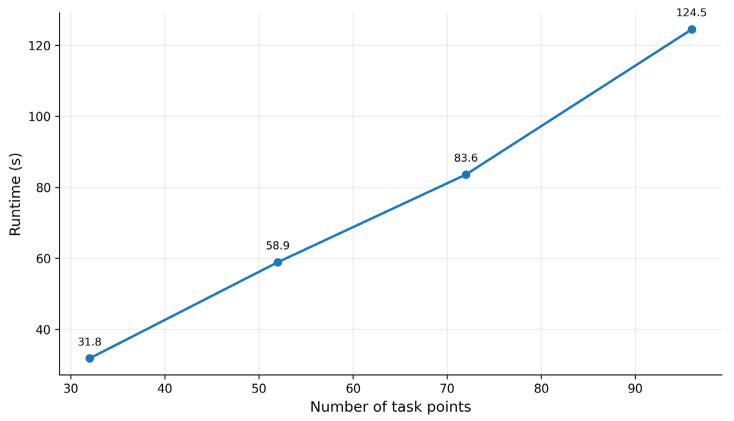
Runtime variation under different task scales.

Figure 12 further illustrates the runtime variation under different task scales. The runtime increases with the number of task points and the scenario size, but the overall growth trend remains relatively smooth. This result indicates that the proposed method has a certain degree of scalability within the current simulation scale. However, if the task scale continues to increase, the number of UAVs further grows, or asynchronous cooperation and more complex uncertain environments are introduced, the search space of the scheduling problem and the difficulty of policy learning will increase substantially. Therefore, future work should further incorporate distributed training, hierarchical task partitioning, and more efficient multi-objective evolutionary mechanisms to improve the applicability of the proposed method to larger-scale real-world inspection tasks.

## 5. Conclusions

To address the challenges of significant vehicle parking constraints, limited UAV endurance, and insufficient multi-task coordination efficiency in distribution network inspection, this paper investigates the task scheduling problem of vehicle–UAV synchronous cooperative power grid inspection, establishes a synchronous cooperative inspection model, and proposes a multi-objective optimization method based on MOTD3-NSGA-II. The main conclusions are summarized as follows.

1. A task scheduling model for vehicle–UAV synchronous cooperative power grid inspection is established. The vehicle–UAV synchronous cooperative inspection process is uniformly modeled from the perspectives of the task-point set, parking-point set, stage-wise vehicle repositioning, UAV launch and retrieval, task completion criteria, and state-of-charge evolution. The coupling relationships among vehicle waiting, UAV stage-wise execution, and energy constraints are characterized, thereby providing a modeling foundation for research on vehicle–UAV synchronous cooperative inspection task scheduling.
2. A multi-objective optimization method for vehicle–UAV synchronous cooperation based on MOTD3–NSGA-II is proposed. With task coverage, mission completion time, minimum residual state of charge, and load balance as the optimization objectives, a multi-objective optimization framework for vehicle–UAV synchronous cooperative inspection is constructed. The outer-layer NSGA-II is used for cooperative parameter search, while the inner-layer MOTD3 is employed for continuous-action policy learning, thereby achieving coordinated optimization among task completeness, execution efficiency, and energy safety.
3. Simulation results verify the effectiveness and adaptability of the proposed method. In four simulation scenarios with different task scales and spatial distribution characteristics, the proposed method achieves 100% task coverage in all cases, with mission completion times of 11,109 s, 9693 s, 10,538 s and 10,721 s, respectively, and minimum residual SOC values of approximately 0.28–0.36. The results show that the proposed method can balance execution efficiency and energy constraints while ensuring inspection completeness, and can maintain good operational stability and scenario adaptability under different scenarios.

It should be noted that the current model in this paper remains a simplified scheduling-level model and still does not fully characterize factors such as random wind disturbances, uncertainty in vehicle road travel conditions, battery degradation, fluctuations in communication states, and execution delays in real inspection environments. Future work will further integrate a more refined UAV energy consumption model, a stochastic vehicle travel time model, and uncertainty-aware robust optimization mechanisms to improve the applicability of the proposed method to complex engineering inspection scenarios.

## Figures and Tables

**Table 1:** Main parameters of the simulation scenarios.

Parameter	Scenario 1	Scenario 2	Scenario 3	Scenario 4
Number of parking points	4	4	4	4
Number of UAVs	4	4	4	4
Number of task points	52	44	48	50

**Table 2:** Overall results of different simulation scenarios.

Scenario	Number of Task Points	Task Coverage	Mission Completion Time/s	Minimum Residual SOC
Scenario 1	52	1.000	11,109	0.2680
Scenario 2	44	1.000	9693	0.3314
Scenario 3	48	1.000	10,538	0.3378
Scenario 4	50	1.000	10,721	0.3584

**Table 3:** Comparison of different scheduling methods under multiple evaluation indicators.

Method	Task Coverage/%	Mission Time/min	Minimum Residual SOC/%	Load Balance Index
Greedy heuristic	92.30	220.25	24.52	0.339
NSGA-II-only	96.60	202.32	28.18	0.296
MOTD3-only	98.10	194.66	28.58	0.276
NSGA-III	98.70	187.92	30.02	0.251
MOEA/D	98.40	190.36	29.64	0.257
MOTD3–NSGA-II	100.00	174.11	31.24	0.230

**Table 4:** Pareto-set quality comparison of different multi-objective methods.

Method	Number of Non-Dominated Solutions	HV	IGD	Spacing
NSGA-II-only	27	0.0628	0.1082	0.0071
NSGA-III	28	0.0822	0.0519	0.0045
MOEA/D	24	0.0819	0.0642	0.0061
MOTD3–NSGA-II	29	0.1018	0.0192	0.0040

**Table 5:** Robustness comparison of different scheduling methods under stochastic perturbations.

Method	Task Coverage/%	Mission Time/min	Minimum Residual SOC/%	Load Balance Index	Full-Mission Success Rate/%
Greedy heuristic	91.41 ± 2.36	233.98 ± 13.26	22.27 ± 3.14	0.370 ± 0.032	63.33
NSGA-II-only	95.70 ± 1.71	208.27 ± 9.11	26.11 ± 2.48	0.318 ± 0.025	76.67
MOTD3-only	97.34 ± 1.22	200.51 ± 7.83	26.75 ± 2.11	0.294 ± 0.018	86.67
NSGA-III	97.94 ± 0.93	193.90 ± 9.08	28.12 ± 1.70	0.267 ± 0.015	93.33
MOEA/D	97.64 ± 1.08	198.50 ± 11.41	27.95 ± 1.83	0.275 ± 0.016	90.00
MOTD3–NSGA-II	100.00 ± 0.00	181.34 ± 7.57	29.73 ± 1.26	0.241 ± 0.010	100.00

**Table 6:** Computational efficiency and scalability results under different task scales.

Scale	Number of Task Points	Number of UAVs	Area Size/km	Runtime/s	Task Coverage/%	Mission Time/min	Minimum Residual SOC/%
Small	32	3	2.5 × 2.0	31.8	100.00	112.6	32.10
Reference	52	4	3.5 × 2.5	58.9	100.00	174.1	31.24
Medium	72	4	4.5 × 3.0	83.6	99.10	236.4	28.70
Large	96	5	5.5 × 4.0	124.5	98.40	303.8	25.60

## Data Availability

The datasets used during the current study are available from the corresponding author upon reasonable request.
